# Awareness and Knowledge of Antibiotic Resistance and Risks of Self-Medication With Antibiotics Among the Aseer Region Population, Saudi Arabia, 2023

**DOI:** 10.7759/cureus.40762

**Published:** 2023-06-21

**Authors:** Muneer J Bhat, Mohammed Al-qahtani, Abdullah S Badawi, Ghufran B Asiri, Abdulaziz M Alhmare, Abdullah Rashid, Khalid S Altalhiyyah, Alhnoof A Alwimny

**Affiliations:** 1 Department of Surgery, King Khalid University, Abha, SAU; 2 College of Medicine, King Khalid University, Abha, SAU

**Keywords:** saudi arabia, practice, attitude, knowledge, population, misuse, self-medication, antibiotic resistance

## Abstract

Background: Antibiotics are a groundbreaking discovery that revolutionized the treatment of infectious diseases in both humans and animals during the 20th century. However, their overuse and misuse led to serious public health threats, causing widespread concern and significant social and economic consequences. Microorganisms have a natural ability to develop resistance to antibiotics over time through genetic mechanisms, which has further exacerbated the problem. Unfortunately, in the last two decades, there has been a dearth of new antibacterial substances discovered, which has only worsened the situation.

Aim: This study aims to assess the awareness and knowledge of antibiotic resistance and risks of self-medication with antibiotics among the Aseer region population, Saudi Arabia 2023.

Methods: An observational cross-sectional survey was conducted in Saudi Arabia targeting persons aged 18 to 80 years old living in the Aseer region, Southern of Saudi Arabia. The data were collected using a pre-structured questionnaire after an intensive literature review and expert's consultation. The questionnaire was distributed throughout the social medial channels. The study questionnaire was uploaded online till no more new cases participated and no new answers were obtained.

Results: A total of 300 participants completed the study questionnaire. Participants' ages ranged from 18 to 80 years with a mean age of 31.5 ± 12.9 years old. Exactly 200 participants (66.7%) were females. As for the educational level, 209 (69.7%) had a university level of education. Two-hundred and three (67.7%) participants had an overall poor knowledge and awareness about antibiotic resistance and risks of self-medication. Also, 103 (34.3%) participants used antibiotics without prescription before and 100 (33.3%) used leftover antibiotics from a previous infection.

Conclusion: In conclusion, the current study showed that nearly one out of three participants in the Aseer region had unprescribed antibiotics. Another unsafe practice was that the same percent used leftover antibiotics from a previous infection. As for participants' knowledge of antibiotic resistance, also one-third of the respondents had good knowledge about the issue.

## Introduction

Antibiotics are drugs created for the prevention and treatment of bacterial diseases by stopping the development of bacteria [1]. Antibiotics have played a crucial role in public health by effectively combating bacterial infections over the years. Despite their success, antimicrobial resistance has become a growing concern due to the ability of pathogenic bacteria to develop resistance to these drugs for various reasons [2,3]. Improper self-medication is a significant issue in low and low-to-middle-income countries, where individuals may lack the necessary knowledge and resources to use medications safely and effectively [4]. Antibiotics are essential treatments in the developing world, particularly where infectious diseases remain a common cause of morbidity and mortality [5].

Self-medication refers to the practice of using medications to treat symptoms or disorders without seeking the advice or guidance of a health care professional. This can involve taking drugs that have not been prescribed, as well as using prescribed medications in a manner that deviates from the recommended dosage or duration of treatment [6]. Antibiotic self-medication is one of the main causes of antibiotic resistance and drug therapy issues, making antibiotic self-medication a global concern. Despite global efforts to limit these practices, antibiotic self-medication continues to be prevalent among developing nations [7-9]. Antibiotic misuse not only leads to higher rates of illness and death, but also decreases the effectiveness of treatments and places a financial burden on patients. Moreover, it creates an environment that promotes the development of drug-resistant bacteria, which can pose a serious threat to public health [10]. Various self-medication practices include obtaining these medications without a prescription from a certified medical practitioner, reusing previous prescriptions, using leftover medications, and sharing them with family members, neighbors, and others [11,12]. The issue of self-medication in Saudi Arabia requires further investigation, as there is a lack of research on this topic from the perspective of the local community [13]. Despite numerous studies on antibiotics misuse and resistance knowledge from various regions around the world, including Saudi Arabia, as far as we know, no studies have been published in the Aseer region on assessing awareness of antibiotic resistance as a consequence of the irrational use of antibiotics, our study aims to measure the awareness and knowledge of antibiotic resistance and risks of self-medication with antibiotics among the Aseer region population, Saudi Arabia, 2023.

## Materials and methods

A questionnaire-based cross-sectional study was conducted targeting Saudi participants aged 18 to 80 years old living in the Aseer region. The study was conducted during the period from 2/4/2023 to 2/6/2023. Participants less than 18 years, non-Saudis, and from outside the Aseer region, who refused to participate in the study, or with incomplete answers were excluded. Data were collected from the participant who met our criteria via electronic data collection (Google Forms) not showing any nominative information that was distributed through social media platforms. The study questionnaire was validated with a pilot study of three experts in the study field and a pilot study of 20 persons to assess tool clarity, validity, and reliability. Assessment was first done independently, and then items with arguments were discussed in detail until having consensus. All suggested changes were applied to improve the questionnaire validity till the final format used in the current study was obtained. The questionnaire is divided into three sections. The first section of the questionnaire included socio-demographics such as age, gender, work, and income. The second section included a closed question regarding knowledge and awareness of antibiotics resistance and self-medication with only one correct answer. The third section included a closed question to detect participants' attitude and practice of self-medication with antibiotics. The eligible persons were asked to fill out the study questionnaire received till no more new answers were obtained. With regard to reliability, the questionnaire trigger items showed a satisfactory level of reliability with Cronbach's Alpha coefficient for scale data of 0.75.

Data analysis

The data were collected, reviewed, and then fed to IBM SPSS Statistics for Windows, Version 21 (Released 2012; IBM Corp., Armonk, New York, United States). All statistical methods used were two-tailed with an alpha level of 0.05 considering significance if the P value was less than or equal to 0.05. Regarding knowledge and awareness, each correct answer was given a one-point score. Overall awareness level regarding antibiotic resistance and risks of self-medication was assessed by summing up discrete scores for different correct knowledge items. The overall knowledge and awareness score were categorized as the poor level if the participants' score was less than 60% of the overall score and good level of knowledge and awareness was considered if the participants' score was 60% or more of the overall score. Descriptive analysis was done by prescribing frequency distribution and percentage for study variables including participants' personal data, health care field work, and monthly income. Also, participants' knowledge and awareness, attitude and practice toward antibiotic resistance, and risks of self-medication were tabulated while the overall knowledge level was graphed. Cross tabulation showed the distribution of participants’ overall knowledge level by their personal data and other factors with the Pearson chi-square test for significance and exact probability test if there were small frequency distributions.

## Results

A total of 300 participants completed the study questionnaire. Participants' ages ranged from 18 to 80 years old with a mean age of 31.5 ± 12.9 years old. Two-hundred participants (66.7%) were females. As for the educational level, 209 (69.7%) had a university level of education, 69 (23%) had below a secondary level of education, and only 10 (3.3%) had a post-graduate level of education. Eighty-one participants (27%) were health care workers, and 162 (54%) had a health care worker in their family. Sixty-eight participants (22.7%) had a monthly income of less than 3000 SR, 98 (32.7%) had a monthly income of 8000-15000 SR, and 68 (22.7%) had a monthly income exceeding 15000 SR (see Table 1).

**Table 1:** Personal data of study participants, Aseer region, Saudi Arabia

Personal data	No	%
Age in years		
18-30	148	49.3%
31-50	116	38.7%
51-80	36	12.0%
Gender		
Male	100	33.3%
Female	200	66.7%
Educational level		
Below secondary	69	23.0%
Secondary	12	4.0%
Diploma / bachelor	209	69.7%
Post-graduate	10	3.3%
Are you a healthcare worker?		
Yes	81	27.0%
No	219	73.0%
Does any family member work in health field?		
Yes	162	54.0%
No	138	46.0%
Income level		
< 3000 SR	68	22.7%
3000-7000 SR	66	22.0%
8000-15000 SR	98	32.7%
> 15000 SR	68	22.7%

In this study, 59.3% of the participants know that antibiotics are used for bacterial infections, 65.3% know that higher doses of antibiotics do not result in faster recovery, and 53% know that antibiotics are not antipyretics as well, 49.3% know that infections originated from AR could enhance risks especially during surgeries or treatments of malignancies, 45.3% know that the resistance of bacteria will render antibiotics ineffective and it will become impossible to treat variant infections, and 44% know that antibiotic resistance is a phenomenon in which drug potency and efficacy are declined and diminished (see Table 2).

**Table 2:** Awareness and knowledge of antibiotic resistance and risks of self-medication with antibiotics among the Aseer region population, Saudi Arabia

Knowledge items	Yes		No		Don't know	
	No	%	No	%	No	%
Are antibiotics used for bacterial infections?	178	59.3%	77	25.7%	45	15.0%
Are antibiotics used for viral infections?	132	44.0%	130	43.3%	38	12.7%
Do you think the antibiotics are antipyretics as well?	101	33.7%	159	53.0%	40	13.3%
Do you think the higher doses of antibiotics result in faster recovery?	58	19.3%	196	65.3%	46	15.3%
Are antibiotics used for Influenza?	133	44.3%	145	48.3%	22	7.3%
Antibiotic resistance is a phenomenon in which drug potency and efficacy is declined and diminished	132	44.0%	38	12.7%	130	43.3%
The resistance of bacteria will render antibiotics ineffective, and it will become impossible to treat variant infections	136	45.3%	71	23.7%	93	31.0%
Me or my family could get harm of antibiotic resistance	124	41.3%	63	21.0%	113	37.7%
Infections originated from AR could enhance risks especially during surgeries or treatments of malignancies	148	49.3%	32	10.7%	120	40.0%

**Figure 1:**
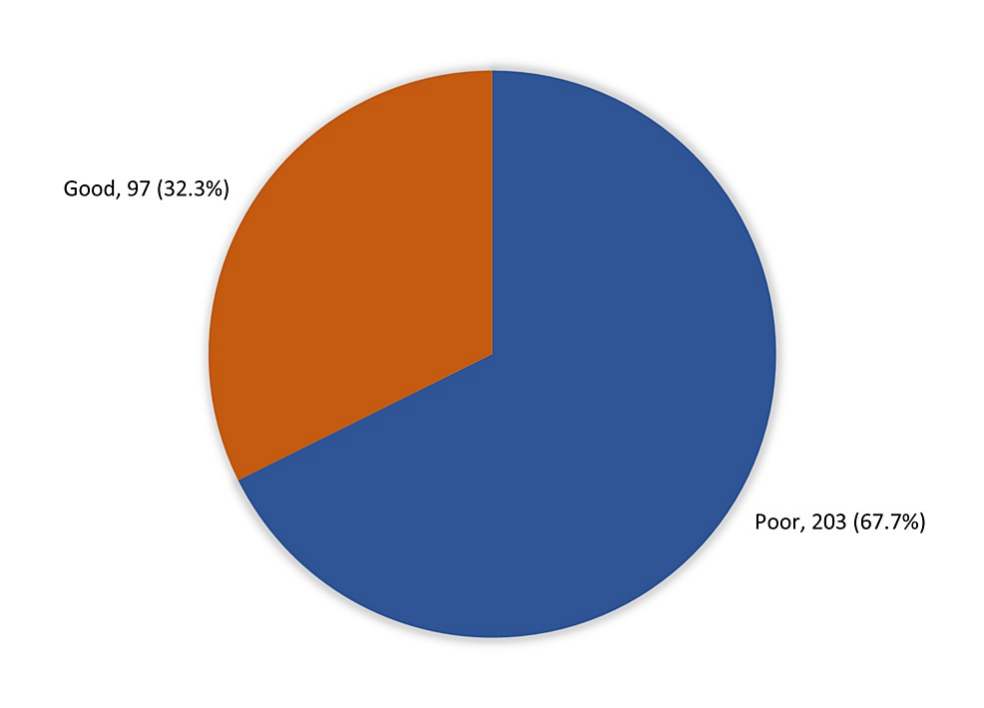
Overall public knowledge and awareness about antibiotic resistance and risks of self-medication with antibiotics among the Aseer region population, Saudi Arabia

Two-hundred and three (67.7%) participants had an overall poor knowledge and awareness about antibiotics resistance and risks of self-medication while 97 (32.3%) had a good knowledge level (see Figure 1).

The vast majority of the study participants (84%) think that self-medication with antibiotics is bad practice, and 198 (66%) think they cannot successfully treat common infectious diseases with antibiotics on their own (see Table 3).

**Table 3:** Participants attitude toward antibiotic resistance and risks of self-medication with antibiotics among the Aseer region population, Saudi Arabia

Attitude items	No	%
What do you think about self-medication with antibiotics?		
Good practice	8	2.7%
Bad practice	252	84.0%
Acceptable practice	40	13.3%
Do you think you can successfully treat common infectious diseases with antibiotics on your own?		
Yes	31	10.3%
No	198	66.0%
Not sure	71	23.7%

One-hundred and three (34.3%) participants used antibiotics without prescription before and 100 (33.3%) used leftover antibiotics from a previous infection. A total of 153 (51%) said that they stopped using antibiotics when they felt better. As for reasons of using antibiotics, the most reported included sore throat (83.3%), fever (47.7%), nasal congestion (38%), diarrhea (28.7%), and skin wounds (27.7%). A total of 255 (85%) reported that they got antibiotics through a doctor prescription. Also, 176 (58.7%) said that they stopped using antibiotics when the course completed and 69 (23%) stopped when symptoms disappeared while 33 (11%) stopped according to doctor opinion (see Table 4).

**Table 4:** Self-medication with antibiotics practice among study participants in the Aseer region, Saudi Arabia UTI: Urinary tract infection

Practice	No	%
Did you use antibiotics without prescription before?		
Yes	103	34.3%
No	197	65.7%
Did you stop using antibiotics when you felt better?		
Yes	153	51.0%
No	147	49.0%
Did you use leftover antibiotics from a previous infection?		
Yes	100	33.3%
No	200	66.7%
Reasons for using antibiotics		
Sore throat	250	83.3%
Fever	143	47.7%
Nasal congestion	114	38.0%
Diarrhea	86	28.7%
Skin wounds	83	27.7%
Cough	72	24.0%
Vomiting	69	23.0%
Aches and pains	59	19.7%
Runny nose	51	17.0%
UTI	8	2.7%
How do you get the antibiotic?		
Doctor's prescription	255	85.0%
Own experience	20	6.7%
Pharmacist opinion	13	4.3%
Dentist prescription	4	1.3%
Friend's opinion	3	1.0%
Advertising	3	1.0%
Family member's opinion	2	.7%
When do you stop using of antibiotic?		
Course completion	176	58.7%
Symptoms disappear	69	23.0%
Doctor's opinion	33	11.0%
Few days after recovery	9	3.0%
Without a cause	5	1.7%
Antibiotics running out	4	1.3%
Pharmacist opinion	4	1.3%

Fifty percent of participants with a post-graduate degree had an overall good knowledge level versus none of those with a secondary level of education with recorded statistical significance (P=.001). Also, 61.7% of health care workers had good knowledge compared to 21.5% of others (P=.001). Good knowledge about antibiotic resistance was assessed among 37.7% of those who think that self-medication with antibiotics is bad practice compared to none of others who think it is good (P=.001). Also, 41.9% of those did not think they can successfully treat common infectious diseases with antibiotics on their own had good knowledge level (P=.001), 36.5% of participants did not use antibiotics without prescription had good knowledge compared to 24.3% of others (P=.031). Likewise, 39.5% of participants who did not use leftover antibiotics from a previous infection had good knowledge about antibiotic resistance versus 18% of those who used (P=.001) (see Table 5).

**Table 5:** Factors associated with public knowledge and awareness regarding antibiotics resistance and risk of self-medication P: Pearson X2 test $: Exact probability test * P < 0.05 (significant)

Factors	Overall knowledge level				p-value
	Poor		Good		
	No	%	No	%	
Age in years					.770
18-30	99	66.9%	49	33.1%	
31-50	81	69.8%	35	30.2%	
> 50	23	63.9%	13	36.1%	
Gender					.727
Male	69	69.0%	31	31.0%	
Female	134	67.0%	66	33.0%	
Educational level					.001*^$^
Below secondary	59	85.5%	10	14.5%	
Secondary	12	100.0%	0	0.0%	
Diploma / bachelor	127	60.8%	82	39.2%	
Post-graduate	5	50.0%	5	50.0%	
Are you a healthcare worker?					.001*
Yes	31	38.3%	50	61.7%	
No	172	78.5%	47	21.5%	
Does any family member work in health field?					.878
Yes	109	67.3%	53	32.7%	
No	94	68.1%	44	31.9%	
Income level					.064
< 3000 SR	49	72.1%	19	27.9%	
3000-7000 SR	52	78.8%	14	21.2%	
8000-15000 SR	60	61.2%	38	38.8%	
> 15000 SR	42	61.8%	26	38.2%	
What do you think about self-medication with antibiotics?					.001*
Good practice	8	100.0%	0	0.0%	
Bad practice	157	62.3%	95	37.7%	
Acceptable practice	38	95.0%	2	5.0%	
Do you think you can successfully treat common infectious diseases with antibiotics on your own?					.001*
Yes	27	87.1%	4	12.9%	
No	115	58.1%	83	41.9%	
Not sure	61	85.9%	10	14.1%	
Did you use antibiotics without prescription before?					.031*
Yes	78	75.7%	25	24.3%	
No	125	63.5%	72	36.5%	
Do you use leftover antibiotics from a previous infection?					.001*
Yes	82	82.0%	18	18.0%	
No	121	60.5%	79	39.5%	

## Discussion

Antibiotic resistance is a growing global public health threat that kills at least 1.27 million people worldwide and is associated with nearly five million deaths annually [14]. One major cause of antibiotic resistance is the irrational overuse of antibiotics, which can result from self-medication [15]. Self-medication is when people use medicines based on their own experience without consulting a doctor [16]. People may use antibiotics for pain management or to cure a disease, and sometimes this may be unnecessary [15]. Such practices can lead to the development of antibiotic-resistant bacteria, which can spread to other people and cause infections that are difficult to treat [17]. It is important to note that antibiotic resistance is not only a problem for individuals who self-medicate but also for the general public and health care providers who may overprescribe antibiotics [18]. Therefore, it is crucial to adopt responsible use of antibiotics and to only use them when prescribed by a health care professional.

The current study aimed to assess the awareness and knowledge of antibiotic resistance and risks of self-medication with antibiotics. With regard to self-medication with antibiotics and its hazards, the current study showed that about one-third of the study participants previously used antibiotics without prescription, and also one-third used leftover antibiotics from a previous infection. The most reported reasons for using antibiotics included sore throat, fever, and nasal congestion (mainly common cold attacks). A higher practice rate was reported in Ethiopia by Simegn et al. who found that prevalence of antibiotics self-medication practice was 55.3% [19]. Also, cough, fever, cold and flu, diarrhea, and headache were the most reported conditions that necessitate antibiotics self-medication. Also, a systematic review and meta-analysis conducted in Africa showed the prevalence of self-medication with antibiotics at 55.7% [20]. Likewise, the current study result was less than other studies conducted in Gondar Town (36.8%) [21], in Ambo (47.9%) [22], in Eritrea (45.1%) [23], Malaysia (15.1%) [24], Sri Lanka (2.6%) [25], and others conducted in different regions [26-29]. In Saudi Arabia, Aldhafar and Talat found that the prevalence of nonprescription antibiotic use was 28.8% which is almost similar to the current study finding [30]. Also, a study conducted in Riyadh by Mohamed et al. showed that 23.6% used nonprescribed antibiotics [31]. The positive finding was that the main reasons for stopping antibiotics were course completion, disappearance of symptoms, and doctor advice which was similar to what was reported by Belkina et al. who showed that 38.8% of the respondents stopped taking antibiotics if they felt better [32].

With regard to participants' awareness regarding antibiotic resistance, the current study showed that about one-third of the respondents were knowledgeable about that issue mainly highly educated, health care workers and those who have good attitude and safe practice toward self-medication. A higher level of awareness was reported in Colombia by Higuita-Gutiérrez et al. who found that 69.3% were aware that empiric antibiotic therapy contributes to antibiotic resistance [33]. Also, in Nigeria, Okedo-Alex found that 64.7% of respondents had good knowledge of antibiotic use and resistance [34]. Similar findings were reported among the Romanian population where 33.3% showed satisfactory knowledge regarding antibiotic resistance and WHO predictions related to this topic [35]. In Saudi Arabia, Aldhafar et al. in Al-Ahsa, Saudi Arabia documented that 61.5% of respondents wrongly believed that antibiotics are used to treat viral infections such as the common cold and influenza [30], and 28.6% of respondents discontinued the antibiotics when they felt better. In Jazan, higher awareness was reported as 56% of the participants knew about antibiotic resistance, with better knowledge about Abs use [36]. Another study conducted in Riyadh in 2017 showed a variation in Abs resistance knowledge among the Saudi population. Approximately 67% did not know what Abs resistance means, 67% denied that Abs can damage children’s teeth, and 65% were unaware that Abs overuse might lead to morbidity and mortality [37]. There are a few limitations to consider when interpreting the findings of this study. Firstly, due to its cross-sectional design, it is not possible to establish a cause-and-effect relationship between sociodemographic factors and participants. Additionally, the study was conducted in a single region, which may not be representative of other regions in Saudi Arabia. Another potential issue is that the data were collected through self-reports, which could be influenced by biases such as social desirability or recall bias. Finally, the study did not examine participants' actual behavior in terms of seeking medical care or adhering to recommendations.

## Conclusions

In conclusion, the current study showed that nearly one out of three participants in the Aseer region had unprescribed antibiotics mainly for flu attacks with congestion which are mainly viral. Also, most of them stopped taking antibiotics with improved symptoms. Another unsafe practice was that the same percent use leftover antibiotics from a previous infection. As for participants' knowledge of antibiotic resistance, also one-third of the respondents had good knowledge about the issue with a considerable portion thinking that antibiotics could be used with viral infection and higher doses associated with faster recovery. Higher education, working in the health care field, and knowing the risky effects of self-medication were associated with a higher knowledge level. More effort should be paid to improve public knowledge and practice toward antibiotic use and its association with antibiotic resistance through specific actions targeted toward the groups of generally low-educated populations where confusion, ambiguity, or misconceptions are mostly documented.
